# Concordance between vocal and genetic diversity in crested gibbons

**DOI:** 10.1186/1471-2148-11-36

**Published:** 2011-02-07

**Authors:** Van Ngoc Thinh, Chris Hallam, Christian Roos, Kurt Hammerschmidt

**Affiliations:** 1 Primate Genetics Laboratory, German Primate Center, Kellnerweg 4, 37077 Goettingen, Germany; 2 WCS Laos, PO BOX 6712, Vientiane, Lao PDR; 3 Gene Bank of Primates, German Primate Center, Kellnerweg 4, 37077 Goettingen, Germany; 4 Cognitive Ethology Laboratory, German Primate Center, Kellnerweg 4, 37077 Goettingen, Germany

## Abstract

**Background:**

Gibbons or small apes are, next to great apes, our closest living relatives, and form the most diverse group of contemporary hominoids. A characteristic trait of gibbons is their species-specific song structure, which, however, exhibits a certain amount of inter- and intra-individual variation. Although differences in gibbon song structure are routinely applied as taxonomic tool to identify subspecies and species, it remains unclear to which degree acoustic and phylogenetic differences are correlated. To trace this issue, we comparatively analyse song recordings and mitochondrial cytochrome b gene sequence data from 22 gibbon populations representing six of the seven crested gibbon species (genus *Nomascus*). In addition, we address whether song similarity and geographic distribution can support a recent hypothesis about the biogeographic history of crested gibbons.

**Results:**

The acoustic analysis of 92 gibbon duets confirms the hypothesised concordance between song structure and phylogeny. Based on features of male and female songs, we can not only distinguish between *N. nasutus*, *N. concolor *and the four southern species (*N. leucogenys, N. siki, N. annamensis*, *N. gabriellae*), but also between the latter by applying more detailed analysis. In addition to the significant correlation between song structure and genetic similarity, we find a similar high correlation between song similarity and geographic distance.

**Conclusions:**

The results show that the structure of crested gibbon songs is not only a reliable tool to verify phylogenetic relatedness, but also to unravel geographic origins. As vocal production in other nonhuman primate species appears to be evolutionarily based, it is likely that loud calls produced by other species can serve as characters to elucidate phylogenetic relationships.

## Background

A striking feature of gibbon songs is the fact that they are species-specific and evolutionarily based. Hence, gibbon songs became a promising tool to identify the taxon affiliation and to describe evolutionary relationships among taxa [1-8]. However, it is also known that gibbon songs are not totally fixed and that they show a certain amount of inter- and a not well-described intra-individual variation [9-12]. Therefore, it remains unclear to what degree acoustic features can be used as taxonomic characters, especially when only subtle differences between populations or taxa are observed. For this, it would be necessary to conduct a systematic comparison of neighbouring populations across different taxa.

In most gibbon species, both males and females sing together [5,13-15], while in a few species males make solo songs in addition to the duets. Until now only two species, the Kloss's gibbon (*Hylobates klossii*) and the silvery gibbon (*H. moloch*), are known where females and males produce solely solo songs [4,6,16,17]. The structure of gibbon songs shows a clear adaptation to improved long-distance transmission. The energy is concentrated in single frequency bands. The frequency of their call elements exhibits only slow modulations and the frequency range of their song syllable lies in an optimized transmission range [18]. With these features, gibbon songs differ from all vocalisations of other nonhuman primates, resembling more songs of typical rainforest birds. It is notable that they are also similar in their proposed functions, like territory advertisement, mate attraction, and strengthening pair bonds [19-25].

**Figure 1:**
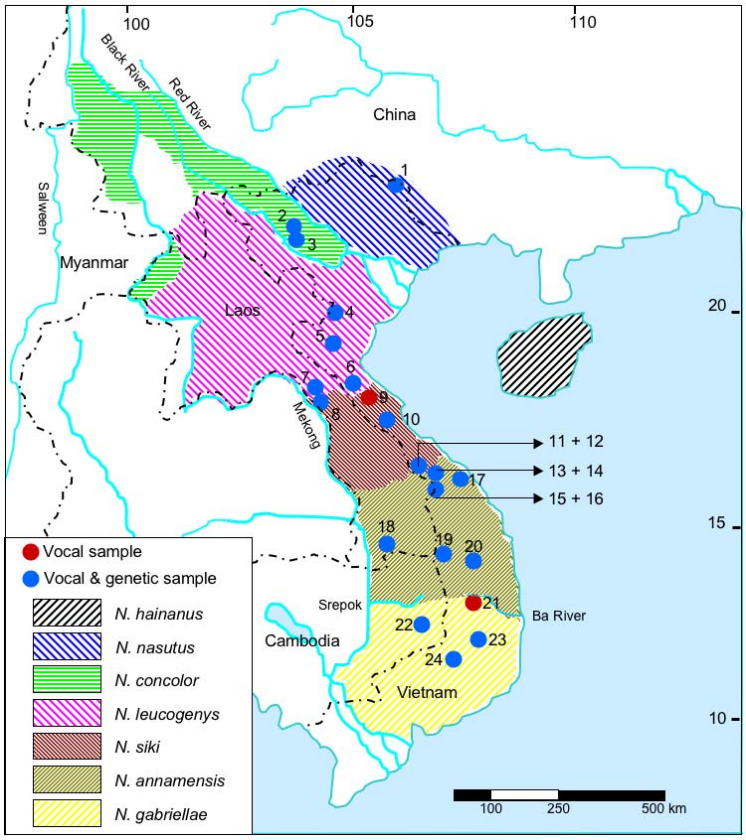
**Geographic distribution of crested gibbons after **[29]. Numbers refer to study populations. For detailed description of recording sites see Additional File 4.

**Figure 2:**
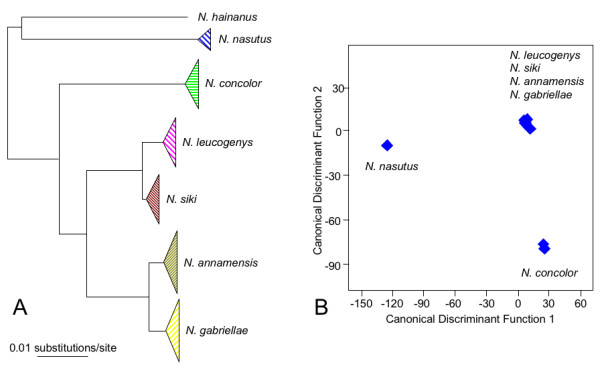
**Phylogenetic relationships among the crested gibbon species (A) and distribution of the six gibbon species based on the scores of the first and second discriminant function (B)**. In A, phylogenetic relationships are based on cytochrome b sequence data and are adapted from [29]. In B, blue diamonds indicate population centroids.

Crested gibbons, genus *Nomascus*, occur only in Vietnam, Laos, Cambodia and parts of southern China (Figure 1). Adults show a strong sexual dichromatism with orange or yellow coloured females, and black males, which in some taxa have light cheeks. The crown hair in males is erect, which gives them their common name "crested gibbons". According to recent investigations using acoustic, genetic and morphological data, seven species of crested gibbons are recognized [8,26-30]. These include the Hainan gibbon (*N. hainanus*), the eastern black gibbon (*N. nasutus*), the western black gibbon (*N. concolor*), the northern white-cheeked gibbon (*N. leucogenys*), the southern white-cheeked gibbon (*N. siki*), the northern buff-cheeked gibbon (*N. annamensis*) and the southern buff-cheeked gibbon (*N. gabriellae*). *N. hainanus *and *N. nasutus *form a clade and are basal among crested gibbons. Among the remaining species, *N. concolor *branched off first, before finally *N. leucogenys/N. siki *and *N. annamensis/N. gabriellae *diverged [28-30] (Figure 2A).

In this study, we aim to elucidate whether differences in song structure of six crested gibbon species and especially of the four closely related species, *N. leucogenys*, *N. siki, N. annamensis *and *N. gabriellae*, are persistent enough to be regarded as phylogenetic trait. Therefore, we recorded more than 400 male and female songs from 92 groups at 24 locations in Vietnam, Laos and Cambodia, and compared the extracted vocal features with gibbon mitochondrial cytochrome b gene sequences from 22 of the 24 populations [29]. Further, we want to describe the relatedness between geographic origin and song similarity as well as genetic distance. We hypothesize that beyond intra- and inter-individual variation, song structure can be used to distinguish between populations and species, and that geographic distance between populations is highly correlated with their song structure.

## Results

### General differences in song structure of crested gibbons

**Figure 3:**
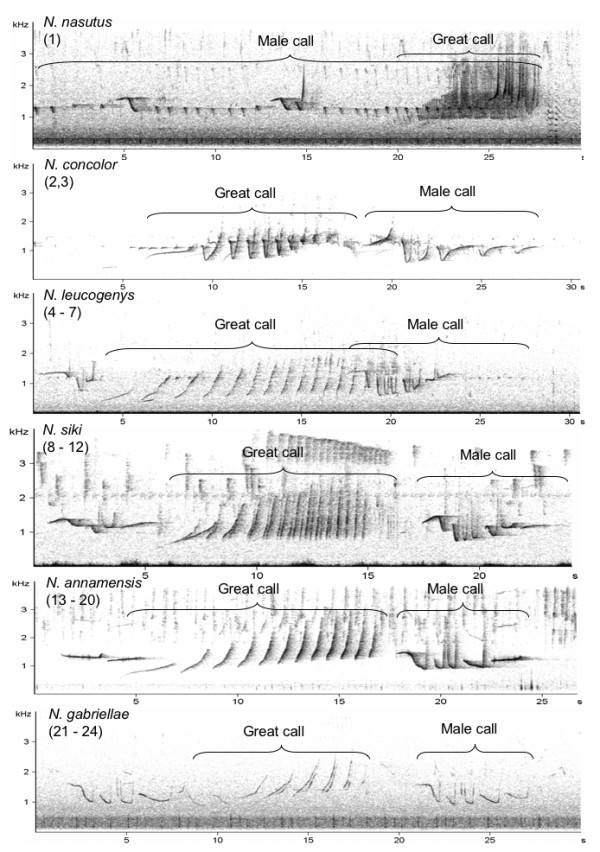
**Spectrograms of the six crested gibbon species analysed in this study**. Numbers in brackets refer to population numbers shown in Figure 1 and Additional File 4.

*N. nasutus *and *N. concolor *could be clearly identified by general acoustic characteristics of their songs (Figure 3, Additional File 1). In contrast, *N. leucogenys*, *N. siki*, *N*. *annamensis *and *N. gabriellae *had very similar song structures and only minor differences could be observed among them (Figure 3).

*N. nasutus *females produced fast up-down sweeps like a spiral spring with a vibrato sound on first two notes. Males produced staccato sounds during, before and after their multi-modulated phrases. All male notes started with almost unmodulated frequency, followed by a down sweep and a fast up sweep. Males of *N. concolor *produced their multi-modulated phrase immediately after the climax of the female great call. The first note of the male call had slightly ascending structure, followed by notes with fast down-up modulation (Figure 3, Additional File 1). Males of *N. leucogenys *gave regularly, loud staccato sounds, which appeared rarely in *N. siki *and *N*. *annamensis*, and were nearly absent in *N. gabriellae*. *N. leucogenys *could be distinguished from the three southern species by their great calls, which had a longer duration and a faster frequency modulation. Accordingly, only population 4 could be assigned to *N. leucogenys *in all criteria, while other *N. leucogenys *populations (5-7) showed criteria which occurred also in *N. siki *and *N*. *annamensis*. *N. siki *populations (8-12) were more similar in their song structure to *N. leucogenys *than to *N*. *annamensis *populations (13-20). The main criteria to distinguish *N. siki *and *N*. *annamensis *songs were criteria 2 and 4 (Additional File 1). In contrast, we found higher similarities between *N. annamensis *populations and *N. gabriellae *(21-24). Here the main criteria were 3 and 5 (Additional File 1).

### Acoustic discrimination of the four southern crested gibbon species

**Figure 4:**
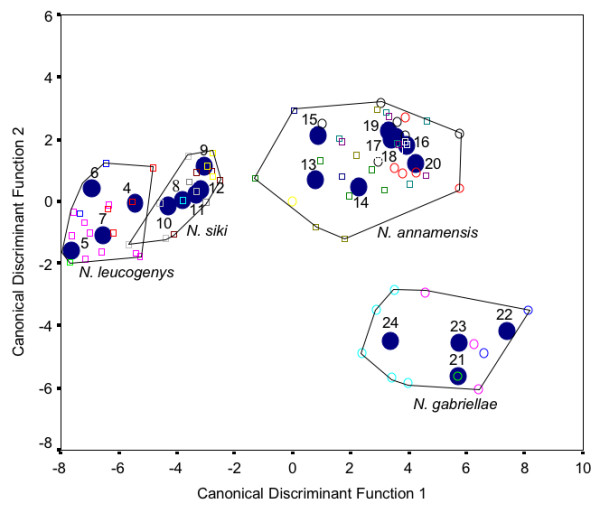
**Distribution of the different populations belonging to the four southern species based on the scores of the first and second discriminant function**. Classification of species based on [29,30] (4 - 7: *N. leucogenys; *8 - 11: *N siki; *12 - 20: *N. annamensis*; 21 - 24: *N. gabriellae*). Blue circles indicate population centroids.

As mentioned above, *N. nasutus *and *N. concolor *could be clearly distinguished from each other and the four southern species, *N*. *leucogenys, N. siki, N. annamensis *and *N. gabriellae*, by general acoustic characteristics, while for latter four species no obvious criteria were found to discriminate among them. Similar results were detected in a first stepwise Discriminant Function Analysis (DFA) (Figure 2B). Accordingly, *N. nasutus*, *N. concolor *and the four southern species formed three clearly differentiated clusters with an assignment of populations to respective clusters of 100%. To discriminate among the four southern species, we performed a second DFA (Figure 4) in which 85.2% of the 81 recorded groups were assigned to its correct species. The assignment accuracy ranged from 50% for population 16, 60% for populations 10 and 17, 75% for populations 14 and 18, 80% for population 13, 90% for population 6, and 100% for the remaining 14 populations. The cross-validation achieved a classification result of 55.6%. The decline in the cross validation is mainly caused by the fact that from some populations we have recordings of only one or two groups. Nevertheless, 55.6% is highly significant above the change level of 4.75%.

The stepwise DFA needed 14 out of the 53 acoustic parameters to achieve this classification result. The DFA included acoustic parameters of both sexes, six parameters of the multi-modulated male call (parameters: 1, 11, 14, 19, 23, 28) and eight parameters of the female great call (parameters: 31, 33, 34, 40, 41, 43, 47, 50; for description see Additional File 2). The scattergram (Figure 4) showed the separation of the 21 populations according to the first and second discriminant function, explaining 54.3% and 12.8% of variation, respectively. The first discriminant function, which mainly represents frequency characteristics of gibbon songs, separated populations 4-12 from populations 13-24. The second discriminant function, which represents temporal features of gibbon songs, separated populations 21-24 from all other populations. Already the first two functions achieved a good separation of the four species with the exception of one group in population 10, which was assigned to *N. leucogenys *instead of to *N. siki*.

We conducted hierarchical cluster analysis (CA) to verify the results of the DFA and to test whether a CA would come up with the same number of expected categories (i.e., species). Based on the acoustic song structure, the CA algorithm revealed a high concordance between the four species (Additional File 3). In total, the CA could correctly classify 68 out of 81 groups (84%). In the first cluster, *N. siki *groups clustered together with groups of *N. leucogenys *(6, 9-14) interspersed by two *N*. *annamensis *groups (33, 34). The second and third cluster comprised only of *N. leucogenys *and *N. gabriellae *groups, respectively. The fourth cluster comprised of *N. annamensis *groups interspersed by three *N. gabriellae *groups (72, 76, 77).

### Correlation between vocal structure, genetic and geographic distance

Among the 21 populations of the four closely related species, *N*. *leucogenys, N. siki, N. annamensis *and *N. gabriellae*, we found a significant correlation between similarity in vocal structure of gibbon songs and geographic distance (Table 1, Additional File 3). A similarly significant correlation was also detected between genetic diversity and geographic distance. To test the concordance between genetic diversity and similarity in song structure, we performed comparisons on species and population level. For the comparison among the four species, a significant positive correlation was observed. Also the comparison of the 19 populations, for which both genetic and acoustic data were available, revealed a significant correlation coefficient.

**Table 1:** Correlation between vocal similarity, genetic and geographic distance*

Distance matrices compared	Populations of collected samples	Rxy	P(rxy-rand > = rxy-data)	Pairwise comparisons
Vocal vs Geographic	21 populations (vocal)	0.672	0.01	190
Genetic vs Geographic	19 populations (genetic and vocal)	0.723	0.01	703
Genetic vs Vocal	19 populations (genetic and vocal)	0.503	0.01	136
Genetic vs Vocal	4 species (genetic and vocal)	0.868	0.02	6

*Rxy = correlation coefficient of Mantel test. P(rxy-rand > = rxy-data) = probability of positive autocorrelation (one tailed).

## Discussion

Acoustic analysis could confirm the concordance between song structure and phylogenetic relationships as obtained from mitochondrial sequence data. We found significant differences between the songs of *N. nasutus*, *N. concolor *and the four southern species, *N. leucogenys*, *N. siki*, *N. annamensis *and *N. gabriellae*. Although latter four species revealed only subtle differences in their songs, a detailed acoustic analysis was able to discriminate significantly between them. This relation was positively correlated with their genetic relatedness found by recent molecular studies [27-32]. In addition, we found a highly significant correlation between similarity in song structure and geographic distance.

Since the early study of Marshall and Marshall [16], we know that gibbon songs are an important trait of their taxonomic relationship [8,10,33]. In many cases, species could be distinguished by directly inspecting the spectrograms of their songs (see Figure 3). However, as can be seen in the same figure, closer related taxa can have very similar song structures. Although there are some studies on individual variation in gibbon songs [9,11], there is no systematic study available to confirm whether individual variation or variation at group level is high enough to contradict a possible relation between song structure and genetic relatedness among closely related species. One reason for this lack of information could be the fact that the comparison of single neighbouring groups revealed unambiguous results. The few misclassifications in our study occurred only between neighbouring groups, whereas groups living far away from each other followed the rule, larger distance dissimilar song structure. It remains undecided whether these misclassifications between neighbouring groups in gibbons have a similar reason as in song birds. In many song bird species, neighbouring males tend to emphasis difference in their vocal repertoire [34]. Song birds seem to use this principle of increased contrast as a tool to settle territory boundaries. However, song birds must learn their songs [35], whereas gibbons have a predominantly innate song structure. Therefore, their ability to produce more distinct songs in relation to their direct group neighbours should be limited. However, we do not know whether gibbons even follow such a rule. The dispersal pattern of males and females could be also an explanation for the misclassification between neighbouring groups. If neighbouring groups are genetically dissimilar then they should also differ in their acoustic structure. Again, we have no precise knowledge about the dispersal patterns of crested gibbons, although at least in a related species, the siamangs, females seem to disperse over longer distances than males [36]. A last possibility is that the misclassification between neighbours is mainly due to the fact that songs of neighbouring groups are too similar and therefore cannot reliably be assigned to the correct group. However, the high significant relation between acoustic similarity, geographic distance and genetic relatedness showed that crested gibbon songs are a salient feature of their genetic relatedness. Accordingly, song structure is a promising tool to identify the taxon-affiliation of gibbon individuals or populations. This is of great importance, because samples from free-ranging gibbons for genetic analyses are difficult to be obtained and fur colouration, especially of female crested gibbons, is due to its extreme intra-taxon variability unreliable [5].

There are not many possibilities to explain our second finding, the high correlation between acoustic structure and geographic distance. The structure of animal vocalisations is under different selective forces, such as body size, habitat characteristic or different functions [37]. However, gibbons are limited to forest habitats and the functions of their songs make it necessary to broadcast them over larger distances. Selection pressure forces under such closed habitat conditions an optimal adaptation of song structure (recently reviewed in [38]). Therefore, the influence of other factors is very limited and gene flow must have achieved the major influence on the structural variation in gibbon songs.

From our acoustic result we cannot conclude the origin of crested gibbons, because we have no evidence, which song structure is most ancestral. However, we found the largest differences in the song structure between the most northern and most southern species with successive gradation between them. This fits very well with the proposed hypothesis that the genus originated in the north and successively migrated to the south [28-30] (Figure 2A).

## Conclusions

In this study, we have shown that in crested gibbons, vocal diversity correlates with genetic relatedness and geographic distance. Accordingly, acoustic analyses provide a reliable tool to support taxon-affiliation and to settle distributional ranges. Furthermore, the acoustic analysis is able to support proposed migration backgrounds. Because other nonhuman primate species have similar constraints in vocal production, it is likely that also in other primate species, loud calls can function as valuable tool to explain taxonomic relations and migration backgrounds.

## Methods

### Survey locations and data collection

In 2007 and 2008, we conducted field surveys in 24 protected areas in Vietnam, Laos and Cambodia (Figure 1, Additional File 4), and recorded songs from *N. nasutus, N. concolor, N. leucogenys*, *N. siki, N. annamensis *and *N. gabriellae*. Recordings in the range of *N. hainanus *were not conducted, but the species clearly differs in song structure from all others [5,6]. Vocalisations were recorded in the early morning using a "listening post" approach based on described methods [39]. When hearing calls, the time and direction was recorded with compass bearings on angle. With this information, it was possible to distinguish calls from different groups. Group positions were depicted on a map (1/50000) to enable changes in listening posts and to ensure the best coverage in obtaining different groups in the observation area. When doubtful whether the same or a nearby group was recorded, the data were excluded from further analysis. We used only 92 out of 175 recordings. In total, we analysed 440 great calls and 447 male calls from 92 different gibbon groups at 24 locations. To record songs, a digital solid state recorder MARANTZ PMD 660; (Marantz, Japan; sampling rate: 44.1 kHz, 16 bit amplitude resolution) and a Sennheiser directional microphone (K6 power module and ME66 recording head with MZW66 pro windscreen; Sennheiser, Wedemark, Germany) was used. Research complied with protocols approved by the National Forest Protection Department of Vietnam, the Cambodian Forestry Administration and the Lao Ministry of Agriculture and Forestry, and adhered to the legal requirements of the countries in which research was conducted. The study was carried out in compliance with respective animal care regulations and the principles of the American Society of Primatologists for the ethical treatment of nonhuman primates.

### Acoustic analysis

Crested gibbon songs consist of phrases from both sexes. Males produce three different phrases including boom, staccato and multi-modulated phrases, and females so-called great call phrases only (Figure 3). For the analysis we considered male phrases as fully developed if they consisted of two or more notes. Female phrases were considered as fully developed if they consisted of six or more notes. The criteria we used to describe the general differences in song structure are listed in Additional File 1.

**Figure 5:**
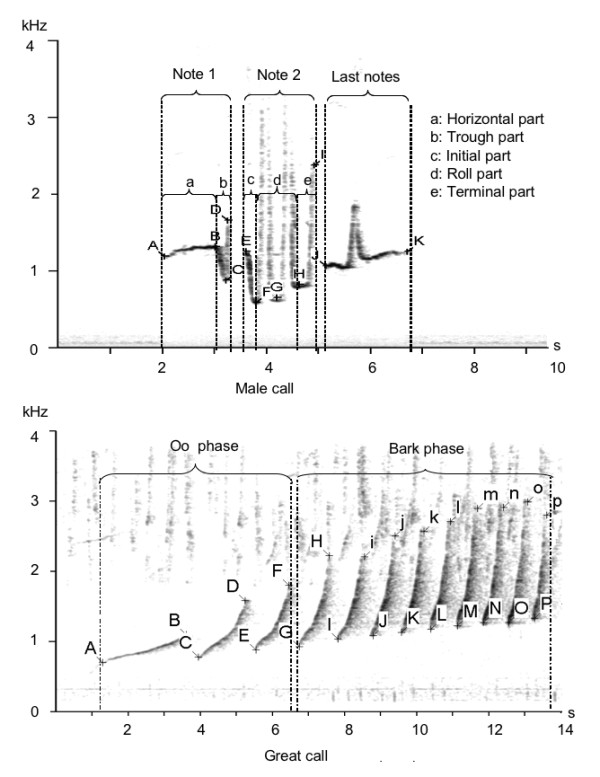
**Spectrogram describing acoustic parameter estimation**. Letters mark points used to calculate acoustic parameter (see also Additional File 2).

We used AVISOFT SASLAB Pro (R. Specht, Berlin, Germany) to generate spectrograms and to calculate acoustic parameters. To find the point with maximum energy at the beginning, ending and anchor points of notes in the frequency spectrum, we used the free reticule cursor tools of AVISOFT (frequency range: up to 500 kHz, frequency resolution: app. 8 Hz, time resolution: 16 ms). In total, we calculated 53 acoustic parameters describing the temporal and frequency structure of male and female gibbon phrases. A detailed description how we measured the acoustic parameters are given in Figure 5. A list with detailed description of the 53 acoustic parameters is given in Additional File 2.

### Statistical analysis

We conducted two DFAs to statistically verify the observations as obtained from the general acoustic descriptions for the six analysed species and to test whether the four southern species, which are not separable by the general acoustic description, can be assigned correctly by the calculated acoustic parameters. We calculated mean values per group using 440 great calls and 447 multi-modulated male calls (population numbers 1-24, see Figure 1, Additional File 4). For the second DFA, including only the four southern species, we used group mean values from 410 great calls and 395 multi-modulated male calls. We analysed a total of 81 different groups from 21 populations (population numbers 4-24, Figure 1, Additional File 4). We standardized the acoustic parameters and conducted all 53 parameters to a stepwise DFA in SPSS 13 [40]. The selection criterion for an acoustic parameter to be entered was *p *= 0.05 and *p *= 0.1 to be removed from the analysis. The assignment of songs to the different populations was cross-validated by the leaving-one-out method [39], which involves leaving out each of the cases in turn, calculating the functions based on the remaining *n*-1 cases and then classifying the left-out case. In addition, we carried out a CA in SPSS to evaluate the similarity in the acoustic structure of the 81 groups. We calculated the z-score variables of the 14 acoustic parameters selected by the stepwise DFA. As distance measure we used the Euclidean distance and cluster method 'between groups linkage'.

To test the statistical relationship between acoustic structure, and genetic and geographic distance matrices, we used a Mantel test as implemented in GenAlex [41]. The vocal distance matrices for the four closely related species, *N*. *leucogenys, N. siki, N. annamensis *and *N. gabriellae*, were generated using the F values of pairwise distances in the stepwise DFA. The geographic distance matrices were calculated from the minimum distance of different groups between the 21 populations. Geographic coordinates were obtained via GPS. Genetic distances were generated using pairwise population F values between haplotypes of mitochondrial cytochrome b gene sequences by the distance function in GenAlex. Respective haplotypes were recently published by our group [28,29] (GenBank: GU595000-GU595004 [*N. leucogenys*], GU321270, GU595005-GU595008 [*N. siki*], GU595009-GU595015 [*N. annamensis*], and GU595017-GU595022 [*N. gabriellae*]).

## Authors' contributions

VNT collected acoustic and genetic samples, did laboratory work, analysed data and wrote the paper, CH collected acoustic and genetic samples, CR designed the study, did laboratory work, analysed data and wrote the paper, KH designed the study, analysed data and wrote the paper. All authors approved the final version of the manuscript.

## Supplementary Material

Additional file 1 **Qualitative criteria to describe crested gibbon species**. Click here for file

Additional file 2 **Descriptions of acoustic parameters used in the DFA **(see Figure 4). Click here for file

Additional file 3 **Dendrogram of hierarchical cluster analysis showing the acoustic dissimilarity between the four southern species**. Click here for file

Additional file 4 **Information about sample locations, molecular identification and number of analysed calls**. Click here for file
