# Supplementary material for: Concordance between vocal and genetic diversity in crested gibbons
Source: BMC Evol Biol. 2011 Feb 7;11:36. doi: 10.1186/1471-2148-11-36 (PMC3044664; doi:10.1186/1471-2148-11-36)
Supplement: Additional file 1 — Qualitative criteria to describe crested gibbon species. [file 1471-2148-11-36-S1.DOC]

Additional File 1: Qualitative criteria to describe crested gibbon taxa.

| Taxon | Male call | Great call | Assigned populations | |
| --- | --- | --- | --- | --- |
| *N. nasutus* | - Booms absent. - Trough part of first note missing in sweep up frequency. No roll spears and initial part of second note start with short sweep up before sweeping down, then rapid changes of frequency modulation up to the last note. - Repeated staccato notes with short and rapid up-down sweeps. - Multi-modulated phrase immediately after first few notes of the great call. | - 8-12 notes and except the first 2-3 very rapid vibrato sounds. - All fundamental frequencies < 2.8 kHz. - Great call elements sweep up-down as spiral spring. | 1-Trung Khanh | |
| *N. concolor* | - Single booms during inflation of throat sac, staccato phrases and multi-modulated phrases. - First note start at high frequency (>1 kHz) and is of ascending, followed by notes with fast up-down modulation. | - 9-14 notes and except the first, ascending frequency only. - From second note fast down-up modulation. | 2-Che Tao  3-Muong La | |
| *N. gabriellae*  *N. siki*  *N. annamensis*  *N. leucogenys* | 1a: Booms during inflation of throat sac.  1b: Booms appears sometime during inflation of throat sac.  1c: Booms absent during inflation of throat sac.  2a: Stable frequency at the beginning with fast down-up sweep at the end.  2b: Starts at low frequency then increasing with a fast down-up-sweep at the end.  2c: Starts low and holds to the end with stable frequency.  3a: Staccato regular.  3b: Staccato not regular.  3c: Staccato rare.  4a: Modulation of rolls slow.  4b: Modulation of rolls fast.  4c: Modulation of rolls very fast.  5a: Rolls on second and third note.  5b: Rolls absent sometime.  5c: Rolls only on second note. | 6a: Series of 9-19 notes and Oo notes <4.  6b: Series of 8-15 notes.  6c: Series of 6-12 notes.  7a: Start frequency of notes low (<600Hz).  7b: Start frequency of notes medium (600Hz-700Hz).  7c: Start frequency of notes high (>700Hz).  8a: Start frequency across all notes constant.  8b: Start frequency across all notes ascending and descending of last few notes.  8c: Start frequency across all notes ascending. | 4-Xuan Lien  5-Pu Huong  6-Vu Quang  7-Nam Kading N  8-Nam Kading S  9-Khe Ve  10-Phong Nha-Ke Bang  11-Huong Hoa  12-Huong Hoa  13-Da Krong  14-Phong Dien  15-Xe Sap  16-Sao La  17-Bach Ma  18-Xe Pian  19-Chu Mom Ray  20-Kon Ka Kinh  21-A Yun Ba  22-Phnom Prich  23-Bi Dup-Nui Ba  24-Ta Dung | 1a, 2a, 3a, 4a, 5a, 6a, 7a, 8a  1a, 2a, 3b, 4a, 5b, 6b, 7b, 8b  1b, 2a, 3b, 4a, 5b, 6b, 7a, 8a  1a, 2a, 3a, 4b, 5b, 6b, 7b, 8a  1b, 2a, 3a, 4b, 5b, 6b, 7b, 8a  1b, 2a, 3b, 4a, 5b, 6b, 7b, 8a  1b, 2a, 3b, 4a, 5c, 6b, 7b, 8b  1b, 2a, 3b, 4a, 5b, 6b, 7b, 8b  1b, 2a, 3b, 4a, 5b, 6b, 7b, 8b  1b, 2b, 3b, 4a, 5c, 6b, 7a, 8b  1b, 2c, 3c, 4a, 5b, 6b, 7b, 8b  1b, 2b, 3c, 4b, 5c, 6b, 7b, 8b  1b, 2b, 3c, 4b, 5c, 6b, 7b, 8b  1b, 2a, 3c, 4b, 5c, 6b, 7b, 8b  1b, 2b, 3c, 4b, 5c, 6c, 7c, 8b  1b, 2a, 3c, 4b, 5c, 6c, 7b, 8b  1b, 2b, 3c, 4b, 5c, 6c, 7c, 8b  1c, 2b, 3c, 4b, 5c, 6c, 7c, 8c  1c, 2b, 3b, 4b, 5c, 6c, 7c, 8c  1c, 2c, 3c, 4b, 5c, 6c, 7c, 8c  1c, 2c, 3c, 4c, 5c, 6c, 7c, 8c |
