# Supplementary material for: Concordance between vocal and genetic diversity in crested gibbons
Source: BMC Evol Biol. 2011 Feb 7;11:36. doi: 10.1186/1471-2148-11-36 (PMC3044664; doi:10.1186/1471-2148-11-36)
Supplement: Additional file 2 — Descriptions of acoustic parameters used in the DFA (see Figure 4). [file 1471-2148-11-36-S2.DOC]

Additional File 2: Descriptions of acoustic parameters used in the DFA. Abbreviations A-P mark the points used to calculate acoustic parameters (see Figure 4).

| No. | Acoustic parameters | Description |
| --- | --- | --- |
|  | Male call |  |
| 1 | Duration of entire male phrase [s] | Time at (J – A) |
| 2 | Duration first note [s] | Time at (D – A) |
| 3 | Relative duration of first notes [%] | No. 2 in % of No. 1 |
| 4 | Duration horizontal part [s] | Time at (B – A) |
| 5 | Relative duration horizontal part [%] | No. 4 in % of No. 2 |
| 6 | Duration trough part [s] | Time at (D – B) |
| 7 | Relative duration trough part [%] | No. 6 in % of No. 2 |
| 8 | Start frequency [Hz] | Frequency at A |
| 9 | Maximum frequency horizontal part (Hz) | Frequency at B/A |
| 10 | Minimum frequency [Hz] | Frequency at C or E or G |
| 11 | Frequency range [Hz] | Frequency at (A – E) |
| 12 | Duration of second note [s] | Time at (H – F) |
| 13 | Relative duration of second notes [%] | No. 12 in % of No. 1 |
| 14 | Duration initial part [s] | Time at (F – E) |
| 15 | Relative duration initial part [%] | No.14 in % of No. 12 |
| 16 | Duration roll part [s] | Time at (G - F) |
| 17 | Relative duration roll part [%] | No. 16 in % of No. 12 |
| 18 | Duration terminal part [s] | Time at (H – G) |
| 19 | Relative duration terminal part [%] | No. 18 in % of No. 12 |
| 20 | Start frequency of second note [Hz] | Frequency at E |
| 21 | Maximum frequency [Hz] | Frequency at E or F or G |
| 22 | Minimum frequency [Hz] | Frequency at E or H or G |
| 23 | Frequency range [Hz] | No. 21 – No. 22 |
| 24 | Minimum frequency initial part [Hz] | Frequency at F |
| 25 | Frequency range initial part [Hz] | Frequency at (E – F) |
| 26 | Frequency range of trough roll part [Hz] | Frequency at (G – F) |
| 27 | Frequency range last trough roll part [Hz] | Frequency at (I - G) |
| 28 | Minimum frequency terminal part [Hz] | Frequency at G |
| 29 | Duration of the last notes [s] | Time at (J - I) |
| 30 | Relative duration of last notes [%] | No. 29 in % of No. 1 |
|  | Great call |  |
| 31 | Duration of entire great call [s] | Time at (p – A) |
| 32 | Number of notes | Total number of elements |
| 33 | Range of start frequencies [Hz] | Frequency at (P – A) |
| 34 | Number of Oo notes | Notes with frequency increase of <=1kHz/s |
| 35 | Duration of Oo phase [s] | Time at (F- A) |
| 36 | Relative duration of Oo phrase [%] | No. 5 in % of No. 1 |
| 37 | Number of bark notes | Notes with frequency increase of >1kHz/s |
| 38 | Duration of bark phase [s] | Time at (p – G) |
| 39 | Relative duration of bark phrase [%] | No.8 in % of No.1 |
| 40 | Duration of first note of Oo phrase [s] | Time at (B – A) |
| 41 | Duration of second note of Oo phrase [s] | Time at (D – C) |
| 42 | Duration of first note of bark phrase [s] | Time at (F – E) |
| 43 | Duration of last note of bark phrase [s] | Time at (p – P) |
| 44 | Frequency range of first note of Oo phrase [Hz] | Frequency at (B – A) |
| 45 | Frequency range of second note of Oo phrase [Hz] | Frequency at (D – C) |
| 46 | Frequency range of third note of Oo phrase [Hz] | Frequency at (F – E) |
| 47 | Frequency range of first note of bark phrase [Hz] | Frequency at (H – G) |
| 48 | First inter-note interval of Oo phrase [s] | Time at (C – B) |
| 49 | Second inter-note interval of Oo phrase [s] | Time at (E – D) |
| 50 | Last inter-note interval of bark phrase [s] | Time at (P – o) |
| 51 | First start freq range between second and first note of Oo phrase [Hz] | Frequency at (C – A) |
| 52 | Second start freq range between first note of bark and last note of Oo [Hz] | Frequency at (G – E) |
| 53 | First start freq range between last and previous note of bark phrase [Hz] | Frequency at F (G - H) |
