# Supplementary material for: Concordance between vocal and genetic diversity in crested gibbons
Source: BMC Evol Biol. 2011 Feb 7;11:36. doi: 10.1186/1471-2148-11-36 (PMC3044664; doi:10.1186/1471-2148-11-36)
Supplement: Additional file 3 — Dendrogram of hierarchical cluster analysis showing the acoustic dissimilarity between the four southern species. [file 1471-2148-11-36-S3.PPT]

## Slide 1
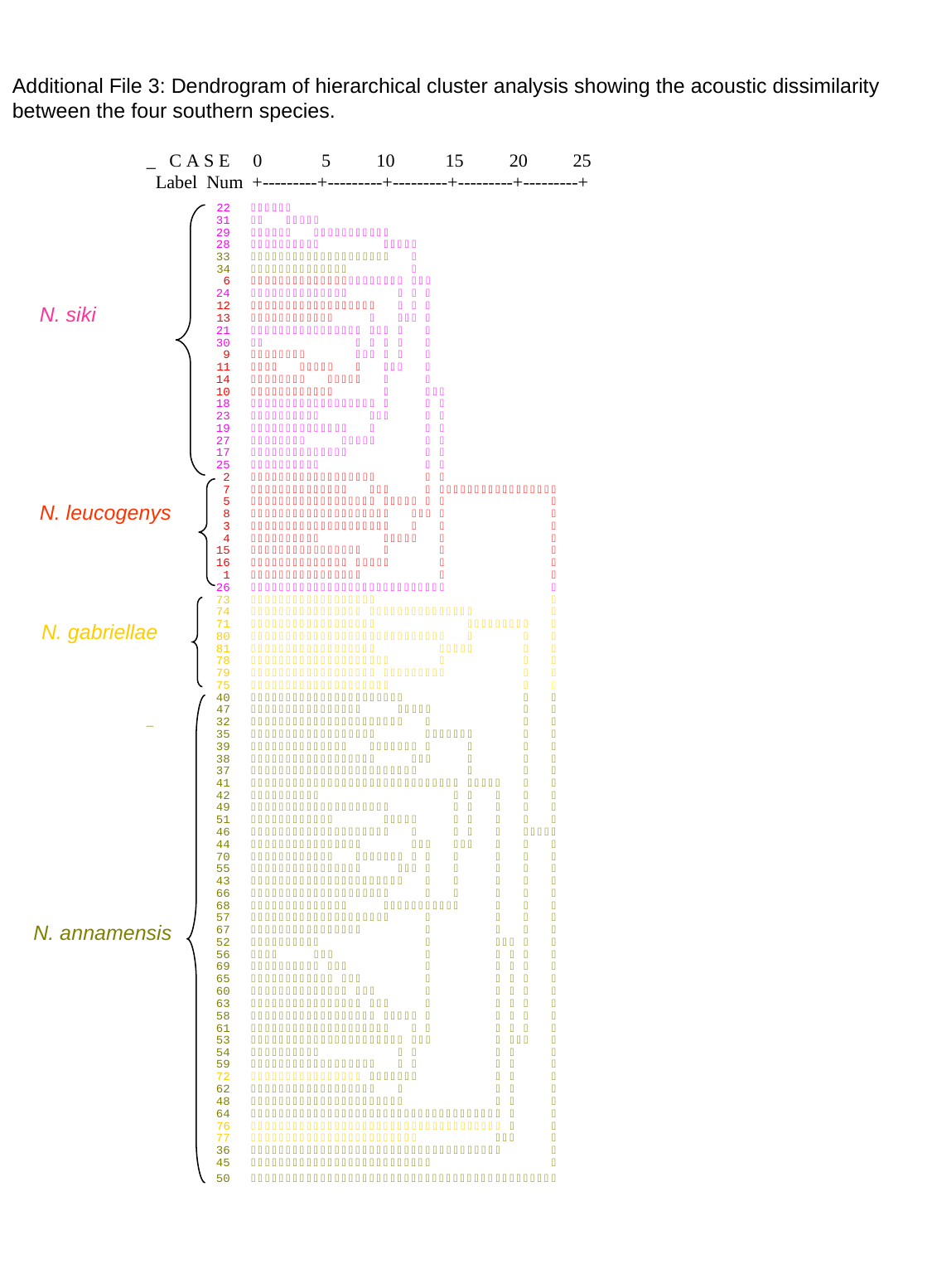

Additional File 3: Dendrogram of hierarchical cluster analysis showing the acoustic dissimilarity between the four southern species.
N. siki
N. leucogenys
N. gabriellae
N. annamensis
