# Supplementary material for: Concordance between vocal and genetic diversity in crested gibbons
Source: BMC Evol Biol. 2011 Feb 7;11:36. doi: 10.1186/1471-2148-11-36 (PMC3044664; doi:10.1186/1471-2148-11-36)
Supplement: Additional file 4 — Information about sample locations, molecular identification and number of analysed calls. [file 1471-2148-11-36-S4.DOC]

Additional File 4: Information about sample locations, molecular identification and number of analysed calls.

| No.* | Samples** | Location | Province, Country*** | | Longitude (N) | Latitude  (E) | Molecular Identification | Recording Time | Analysed Groups | Great Calls | Male Calls |
| --- | --- | --- | --- | --- | --- | --- | --- | --- | --- | --- | --- |
| 1 | v + g | Trung Khanh | Cao Bang, VN | | 22° 51’ 10’’ | 106° 42’ 58’’ | *N. nasutus* | 09/2007 | 5 | 13 | 26 |
| 2 | v + g | Che Tao | Yen Bai, VN | | 21° 42’ 30’’ | 104° 06’ 26’ | *N. concolor* | 07/2007 | 2 | 9 | 14 |
| 3 | v + g | Muong La | Son La, VN | | 21° 35’ 14’’ | 104° 16’ 18’’ | *N. concolor* | 10/2008 | 4 | 8 | 12 |
| Populations 1-3 can be distinguished by quantitative analyses | | | | | | | |  | **11** | **30** | **52** |
| 4 | v + g | Xuan Lien | | Thanh Hoa, VN | 19° 57’ 01’’ | 105° 00’ 18’’ | *N. leucogenys* | 06/2007 | 4 | 14 | 17 |
| 5 | v + g | Pu Huong | | Nghe An, VN | 19° 21’ 42’’ | 104° 56’ 02’’ | *N. leucogenys* | 12/2007 | 1 | 2 | 2 |
| 6 | v + g | Vu Quang | | Ha Tinh, VN | 18° 16’ 29’’ | 105° 26’ 35’’ | *N. leucogenys* | 06/2008 | 2 | 11 | 12 |
| 7 | v + g | Nam Kading (N) | | Bolikhamxai, Laos | 18°39’ 00’’ | 104° 26’ 07’’ | *N. leucogenys* | 2007 | 7 | 36 | 40 |
| 8 | v + g | Nam Kading (S) | | Bolikhamxai, Laos | 18°18’ 45’’ | 104° 26’ 57’’ | *N. siki* | 2007 | 3 | 9 | 16 |
| 9 | v | Khe Ve | | Quang Binh, VN | 17° 54’ 08’’ | 105° 46’ 40’’ | no data | 06/2008 | 3 | 12 | 14 |
| 10 | v + g | Phong Nha-Ke Bang | | Quang Binh, VN | 17° 29’ 09’’ | 106° 21’ 10’’ | *N. siki* | 08/2007 | 5 | 25 | 34 |
| 11 | v + g | Huong Hoa | | Quang Binh, VN | 16° 59’ 28’’ | 106° 35’ 59’’ | *N. siki* | 07/2008 | 2 | 17 | 17 |
| 12 | v + g | Huong Hoa | | Quang Tri, VN | 16° 55’ 49’’ | 106° 35’ 45’’ | *N. siki* | 07/2008 | 4 | 17 | 24 |
| 13 | v + g | Da Krong | | Quang Tri, VN | 16° 24’ 40’’ | 107° 05’ 26’’ | *N.* sp. | 10/2007 | 5 | 24 | 13 |
| 14 | v + g | Phong Dien | | Thua Thien-Hue, VN | 16° 24’ 22’’ | 107° 10’ 01’’ | *N.* sp*.* | 10/2007 | 4 | 19 | 18 |
| 15 | v + g | Xe Sap | | Sekong, Laos | 16° 04’ 04’’ | 107° 15’ 04’’ | *N.* sp*.* | 08/2008 | 2 | 15 | 11 |
| 16 | v + g | Sao La | | Thua Thien-Hue, VN | 16° 06’ 46’’ | 107° 26’ 34’’ | *N.* sp*.* | 08/2008 | 4 | 21 | 15 |
| 17 | v + g | Bach Ma | | Thua Thien-Hue, VN | 16° 12’ 03’’ | 107° 44’ 45’’ | *N.* sp*.* | 11/2007 | 5 | 23 | 24 |
| 18 | v + g | Xe Pian | | Champasak, Laos | 14° 34’ 46’’ | 106° 08’ 04’’ | *N.* sp*.* | 10/2008 | 5 | 27 | 18 |
| 19 | v + g | Chu Mom Ray | | Kon Tum, VN | 14° 25’ 56’’ | 107° 42’ 47’’ | *N.* sp*.* | 11/2007 | 8 | 53 | 33 |
| 20 | v + g | Kon Ka Kinh | | Gia Lai, VN | 14° 20’ 20’’ | 108° 24’ 50’’ | *N.* sp*.* | 09/2008 | 6 | 32 | 20 |
| 21 | v | A Yun Pa | | Gia Lai, VN | 13° 18’ 59’’ | 108° 22’ 05’’ | no data | 08/2009 | 1 | 6 | 4 |
| 22 | v + g | Phnom Prich | | Mondulkiri, Cambodia | 12° 44’ 37’’ | 107° 01’ 54’’ | *N. gabriellae* | 12/2008 | 3 | 17 | 24 |
| 23 | v + g | Bi Dup-Nui Ba | | Lam Dong, VN | 12° 11’ 37’’ | 108° 41’ 06’’ | *N. gabriellae* | 12/2007 | 5 | 19 | 20 |
| 24 | v + g | Ta Dung | | Dak Lak, VN | 11° 52’ 51’’ | 107° 57’ 27’’ | *N. gabriellae* | 11/2008 | 2 | 11 | 19 |
| Populations 4-24 can not be distinguished by quantitative analyses | | | | | | | |  | **81** | **410** | **395** |
| **Total** | | | | | | | |  | **92** | **440** | **447** |

Location numbers refer to those shown in Figure 1; ** v: vocal samples, g: genetic samples; *** VN: Vietnam
